# Uncertainty aware domain incremental learning for cross domain depression detection

**DOI:** 10.1038/s41598-025-10917-y

**Published:** 2025-07-14

**Authors:** Zita Lifelo, Jianguo Ding, Huansheng Ning, Sahraoui Dhelim

**Affiliations:** 1 School of Computer and Communications Engineering, University of Science and Technology Beijing, Beijing, 100083 China; 2 Department of Computer Science, Blekinge Institute of Technology, 371 79 Karlskrona, Sweden; 3 School of Computing, Dublin City University, Dublin 9, Ireland

**Keywords:** Major depressive disorder, Data-free domain alignment, Incremental learning, Class imbalance, Uncertainty estimation, Psychology, Mathematics and computing

## Abstract

Deep learning techniques have demonstrated significant promise for detecting Major Depressive Disorder (MDD) from textual data but they still face limitations in real-world scenarios. Specifically, given the limited data availability, some efforts have resorted to aggregating data from different domains to expand the data volume. However, these approaches face critical challenges, including data privacy, domain gaps, class imbalance, and uncertainty arising from both the data and the model. To overcome these challenges, we propose an Uncertainty-Aware Domain Incremental Learning framework for Cross-Domain Depression Detection (UDIL-DD), integrating Uncertainty-guided Adaptive Class Threshold Learning (UACTL) and Data-Free Domain Alignment (DFDA). Specifically, our UACTL module measures the discrepancy between predictions across sequential domains and learns adaptive thresholds tailored to each class, incorporating predictive uncertainty to enhance robustness. Subsequently, the DFDA module leverages domain-similar samples identified by UACTL to approximate historical feature distributions without accessing previous domain data, effectively addressing catastrophic forgetting. To validate the effectiveness of the proposed method, we conduct extensive experiments on four benchmark MDD datasets-CMDC, DIAC-WoZ, MODMA and EATD confirming the effectiveness of our method’s potential for reliable depression detection in real-world clinical scenarios.

## Introduction

Major Depressive Disorder (MDD) is a significant psychiatric condition affecting millions globally, across various languages and cultures. This condition is marked by a persistent low mood and is frequently associated with diminished interest in life and social activities^1,2^. Accurate and timely diagnosis is critical for effective interventions, reducing severe outcomes like disability and suicide^3,4^. However, traditional diagnostic methods used in clinical practice remain highly subjective, influenced by clinician biases, patient interactions, and environmental factors^3,5^.

Consequently, researchers have employed deep learning approaches utilising physiological signals^6,7^, such as text, audio, video, and EEG, to yield more objective, consistent, and reliable evaluations of MDD. While the results are encouraging, existing deep learning methods for MDD detection are significantly reliant on the presence of large labelled datasets. In clinical practice, gathering adequate labelled data presents significant challenges due to factors like patient privacy, higher collection costs, and restricted institutional collaboration, leading to fragmented datasets that hinder the effectiveness of deep learning techniques^7^. One plausible strategy to address data scarcity involves aggregating datasets across multiple healthcare institutions^7–9^. Nevertheless, directly combining these datasets is problematic, as variations in feature distributions, attributed to differences in culture, language, socioeconomic factors, and institutional practice, often cause significant domain shifts. Such domain shifts significantly hinder models’ capacity to generalise effectively across varied patient populations.

While methods for Domain Adaptation (DA) have been developed to address these discrepancies^5,10^, they often encounter significant obstacles, such as semantic misalignment across domains, increased computational complexity, and the risk of catastrophic forgetting, which can lead to notable performance decline when sequentially applied to new domains^11,12^. Moreover, training multiple models to accommodate different feature distributions is notably time-consuming and impractical. Therefore, it is crucial to create a universal model that can generalise effectively across various domains, instead of training separate models for each dataset.

To effectively tackle the challenges linked to domain shifts and catastrophic forgetting, recent studies have investigated Domain Incremental Learning (DIL) frameworks. These frameworks incrementally adapt a single model to integrate new data domains as they arise, all the while maintaining knowledge from domains that have been learnt previously^13,14^. Traditional DIL approaches generally rely on continuous access to historical raw data, allowing models to refer back to previous distributions throughout the training process^15,16^. In practical clinical environments, the availability of historical patient data is significantly restricted because of strict privacy regulations and ethical issues surrounding the sharing of sensitive medical records. As a result, practical applications in clinical settings typically offer access to trained models or technical resources instead of raw patient data, which greatly restricts the effective implementation and generalisation capability of conventional DIL methods^7^.

To address these challenges, methods for Data-Free Domain Incremental Learning (DF-DIL) have been developed, focusing on maintaining previously acquired knowledge without depending on historical data by creating synthetic samples that closely resemble historical distributions^17,18^. Nonetheless, these existing DF-DIL techniques typically depend heavily on known statistical properties or prior domain knowledge, rendering them impractical in clinical scenarios where such historical statistical information is unavailable or incomplete^7^. Building on this, recent work has introduced a DF-DIL framework specifically for MDD detection, which incorporates a domain alignment mechanism that operates without access to raw historical data^7^, representing a significant step toward practical deployment under real-world constraints.

Class imbalance, while a notable challenge in DIL for MDD detection, has not been thoroughly examined, despite its crucial role in influencing model performance. Clinical datasets typically demonstrate an uneven distribution of classes, characterised by a lower number of samples from the minority class, which usually consists of individuals diagnosed with MDD. Many incremental learning methods utilise a consistent threshold for identifying high-confidence predictions, which unintentionally prioritises the majority class and overlooks instances from the minority class^5^. The application of uniform selection thresholds results in the under-representation of minority-class samples, which in turn introduces biases favouring majority classes during the subsequent training phases. This cyclic effect exacerbates class imbalance throughout incremental learning stages, leading to a decline in the reliability of clinical predictions and an increase in model bias, resulting in a substantial decrease in performance on minority classes^19,20^. While certain approaches have attempted to address the imbalance issue by utilising unlabelled or synthetic data, this adds layers of complexity that increase predictive uncertainty^21,22^, potentially compromising the reliability and integrity of automated MDD detection systems.

Reliable uncertainty quantification is essential for addressing these limitations. Evidential Deep Learning (EDL) has recently emerged as an effective approach for explicitly modelling uncertainty, especially in scenarios where the model lacks sufficient evidence to make confident predictions^23–25^. This method has demonstrated effectiveness in identifying out-of-distribution (OOD) data, where incoming data significantly diverges from previously encountered instances^26^. Managing OOD data is crucial in clinical settings, where data is subject to continuous change and cannot be presumed to maintain consistency over time. Recent evidence indicates that managing uncertainty is essential for enhancing a model’s capacity to generalise across varied and unseen data distributions^24^, reinforcing the value of incorporating EDL into DIL frameworks.

To this end, we propose a novel framework, termed Uncertainty-aware Domain Incremental Learning framework for cross-domain Depression Detection (UDIL-DD), which integrates evidential uncertainty estimation into a DIL setting for depression detection from clinical transcripts. Specifically, we incorporate evidential deep learning into our classifier to estimate predictive confidence using Dirichlet distributions, enabling principled uncertainty modeling under evolving domain shifts. Building on this, we design an Uncertainty-guided Adaptive Class-specific Threshold Learning (UACTL) module that dynamically learns soft thresholds for each class based on the divergence between current and historical predictions, allowing the model to identify and retain low-uncertainty samples from both majority and minority classes. Unlike prior approaches that rely on fixed or manually derived thresholds, this module adapts in real-time to class distribution changes across domains.

To mitigate catastrophic forgetting in a data-free setting, we further introduce a Data-Free Domain Alignment (DFDA) module that partitions current data into domain-similar and domain-dissimilar subsets using uncertainty-aware criteria. We then apply a domain alignment constraint based on Maximum Mean Discrepancy (MMD) to reduce distributional gaps between these subsets, thereby addressing the challenges of data scarcity and privacy by approximating historical feature patterns without access to raw data. Together, these components establish a robust, uncertainty-aware framework that facilitates knowledge retention, improves generalization, and enhances reliability in incremental MDD detection across diverse clinical domains.

In summary, our key contributions are as follows:

- We propose UDIL-DD, an incremental learning framework for MDD detection that integrates uncertainty estimation into a data-free, domain-incremental setting using clinical transcripts. The framework unifies selective learning, prediction, and knowledge retention. Our method consistently improves performance, demonstrating its ability to balance the trade-off between sensitivity and specificity across diverse clinical domains.
- We introduce an Uncertainty-guided Adaptive Class-specific Threshold Learning module that incorporates evidential uncertainty to dynamically adapt thresholds specific to each class. Compared with other threshold methods, UACTL has been shown to significantly boost recall and reduce prediction bias against minority classes, thereby mitigating class imbalance and enhancing overall performance.
- We propose a Data-Free Domain Alignment (DFDA) module that approximates prior domain distributions through sample partitioning and MMD-based alignment, preserving knowledge without accessing historical data. Experimental analyses indicate that this approach leads to substantially lower forgetting scores, consistent performance retention over sequential learning tasks and OOD detection, crucial for real-world clinical applications.

The remainder of this paper is structured as follows: In section “Related work” reviews related work on MDD detection, incremental learning, and uncertainty estimation. Section “Method” details our proposed methodology while section “Experiments” describes the datasets, experimental setups and comparison methods. Section “Results and discussion” presents our findings, along with an analysis of limitations and suggestions for future research. Finally, section “Conclusion” concludes the paper.

## Related work

### Major depressive disorder detection

Recent advancements in machine learning have enabled the development of efficient tools for the automated detection of Major Depressive Disorder (MDD), primarily leveraging textual and auditory indicators that reflect a patient’s mental state^1,5,27^. Traditional techniques have relied on manually crafted features combined with standard classifiers such as support vector machines or logistic regression; nonetheless, these methods often fail to sufficiently capture the intricate and high-dimensional characteristics of natural language and speech data. The rise of deep learning has resulted in improved performance of models such as RNNs and CNNs, which adeptly capture temporal and semantic patterns from text and audio^2,28^. For instance, textual features like negative sentiment and the use of first-person pronouns are associated with the severity of depression. Additionally, acoustic markers, including mel-cepstral coefficients and formants, reflect emotional states^2^.

Recently, transformer-based pre-trained language models (PLMs), including BERT^29^ and its variants, have emerged as the predominant architecture for text representation, enabling the extraction of generalised semantic embeddings even in low-resource contexts^30^. PLMs have shown impressive capabilities in identifying depression through textual interviews and self-reported narratives^4,31^. A notable obstacle for text-based models lies in their ability to generalise. Studies indicate that models for detecting depression, when trained on a single dataset, frequently struggle to adapt to different datasets because of variations in domain-specific language, resulting in diminished performance^4^. This presents a challenge in domain adaptation within NLP, marked by considerable differences in textual distributions among various sources. Current models frequently exhibit robust performance within their specific training domains; nevertheless, they generally show limited adaptability when it comes to generalising to new, unseen domains. This study addresses this limitation by focusing on cross-domain robustness in text-based MDD detection, thereby ensuring that the learned representations are semantically rich and capable of adapting to various clinical data sources.

### Incremental learning

Incremental Learning (IL), also referred to as continual or lifelong learning, is a paradigm that enables machine learning models to sequentially acquire new knowledge over time while preserving previously learned information^16^. This approach is particularly valuable in dynamic environments where data distributions evolve continuously, closely mirroring human learning processes^32^. In contrast to traditional static learning setups, IL aims to ensure both learning plasticity and memory stability, although achieving this balance remains a central challenge due to the risk of catastrophic forgetting^16^. Depending on the nature of the data and learning objectives, IL can be categorized into task-incremental^33,34^, class-incremental^21,35,36^, and domain-incremental learning^37,38^. In task-incremental learning, models are trained on distinct tasks with isolated objectives and benefit from task identifiers during inference. Class-incremental learning introduces new categories over time, requiring the model to discriminate among all classes seen so far without task-specific information. Domain-incremental learning focuses on adapting to shifting feature distributions across domains while maintaining performance on previously learned domains.

Various approaches have been developed to address forgetting in IL systems: replay-based methods^35,39,40^, regularization-based^41^, knowledge distillation^18,42^ and parameter isolation^43^ approaches. Replay-based methods store and replay subsets of past data or generated samples to reinforce prior knowledge. Regularization-based methods mitigate catastrophic forgetting by imposing constraints on parameter updates via loss penalties, which helps maintain the integrity of weights essential for previously learnt tasks. Knowledge distillation synchronises predictions or intermediate representations of current models with those of previous models to ensure consistency throughout learning sessions. Other approaches employ parameter isolation strategies, assigning different parts of the network to particular tasks or domains. Replay and regularisation techniques have become increasingly important because they offer a favourable combination of efficiency and effectiveness, which makes them especially appropriate for practical applications. Nonetheless, numerous traditional IL methods function based on the premise that historical data is available for retention and reuse. This assumption lacks practicality in clinical settings where mental health data is sensitive, governed by stringent privacy regulations, and frequently not transferable between institutions^7^. These constraints have led to the emergence of data-free incremental learning strategies that avoid storing raw data, instead relying on model outputs or distributional approximations to preserve historical knowledge. Our approach centres on identifying domain-representative samples from the existing data that exhibit similar distributional features. This enables the model to align with prior knowledge and reduce the risk of forgetting, all while adhering to data privacy regulations.

### Uncertainty estimation

Estimating uncertainty is crucial for enhancing the robustness of models, particularly in clinical and continual learning environments. Two main categories of uncertainty are typically identified: aleatoric uncertainty, which originates from noise in the data, and epistemic uncertainty, which stems from insufficient data or gaps in knowledge^44^. Bayesian methods and ensemble approaches are commonly employed to model epistemic uncertainty; however, they frequently face significant computational costs^25^. Recent work has demonstrated effective alternatives for uncertainty modelling in specialised domains focussing on confidence calibration; for example, Xu et al.^45^ propose a calibrated one-class classification framework to model predictive uncertainty in unsupervised time series anomaly detection, reinforcing the importance of well-calibrated predictive confidence. In affective computing, uncertainty techniques have emerged for emotion recognition applications. Prabhu et al.^46^ leveraged Bayesian neural networks to model label subjectivity in emotion recognition, while Wu et al.^47^ proposed deep evidence regression to jointly capture aleatoric and epistemic uncertainties. Other methods include calibrated ordinal latent distributions for multimodal fusion^48^ and pairwise uncertainty estimation to address annotation ambiguities^49^. However, these methods face limitations in depression contexts due to: (1) heavy reliance on large annotated datasets rarely available for depression, and (2) absence of statistically rigorous uncertainty guarantees critical for clinical deployment.

Recently, Li et al.^50^ introduced conformal depression prediction (CDP), which overcomes these limitations via distribution-free confidence intervals with theoretical coverage guarantees. Unlike prior approaches, CDP operates as a plug-and-play module requiring no model retraining. Although CDP excels in providing marginal coverage, our work adopts evidential deep learning (EDL) because of its complementary strengths. EDL utilises subjective logic to explicitly model predictive uncertainty during training, by facilitating the explicit estimation of the model’s confidence in its predictions^23^. EDL is particularly suited for tasks involving OOD detection and open-set recognition, where capturing low-confidence or ambiguous predictions is crucial. It offers the ability to separately model situations where the model lacks evidence and where conflicting evidence is present, making it especially effective in non i.i.d.^25^, evolving data environments typical of continual learning scenarios. Recent works have further extended EDL to semi-supervised learning^51^, multimodal inference^24^, and continual learning^25,52^, where uncertainty is used to guide sample selection or to regularize network updates, thereby mitigating catastrophic forgetting. In our study, we adopt evidential uncertainty estimation to enhance domain-incremental MDD detection, enabling the model to distinguish reliable from unreliable predictions and selectively retain knowledge across evolving domains.

## Method

All methods were carried out in accordance with relevant guidelines and regulations. Written informed consent was obtained from all participants prior to the experiments. The study protocols and consent forms were approved by the appropriate institutional ethics committees, and all methods were carried out in accordance with the Declaration of Helsinki. Further details, including dataset-specific ethical approvals and consent information, are provided in the Experiment section under Datasets.

In this section, we introduce our incremental learning framework for cross-domain depression detection. Our framework integrates three key components: a Feature Preprocessing Module that leverages mBERT to extract contextualized embeddings, an Uncertainty-guided Adaptive Class Threshold Learning (UACTL) Module to robustly address class imbalance and select reliable samples, and a Data-Free Domain Alignment (DFDA) Module that mitigates catastrophic forgetting without accessing raw historical data. We begin by formulating the problem and outlining our learning targets, then provide an overview of the framework, and finally detail each component of our method.

### Task definition

We consider a sequence of domains denoted as $\mathscr {D} = \{D^{(i)}\}_{i=1}^{n}, \, i \in [1,n]$, representing $n$ domains. Each domain $D^{(i)}$ is defined as a tuple $D^{(i)} = (X^{(i)}, Y^{(i)})$, where $X^{(i)} \in \mathbb {R}^{Q_i \times d}$ denotes the feature matrix for the $i$-th domain. Each of the $Q_i$ samples is represented by a $d$-dimensional feature vector $X_{i,j}$, where $j$ denotes the index of the $j$-th sample in domain $i$. Correspondingly, $Y^{(i)} \in \mathbb {R}^{Q_i \times K}$ denotes the label matrix, with each row $Y_{i,j}$ indicating the label vector across $K$ classes. We set $K = 2$, where the label $m$ denotes a positive instance and $h$ denotes a negative instance. The label space remains consistent across all domains.

### Proposed method

**Fig. 1:**
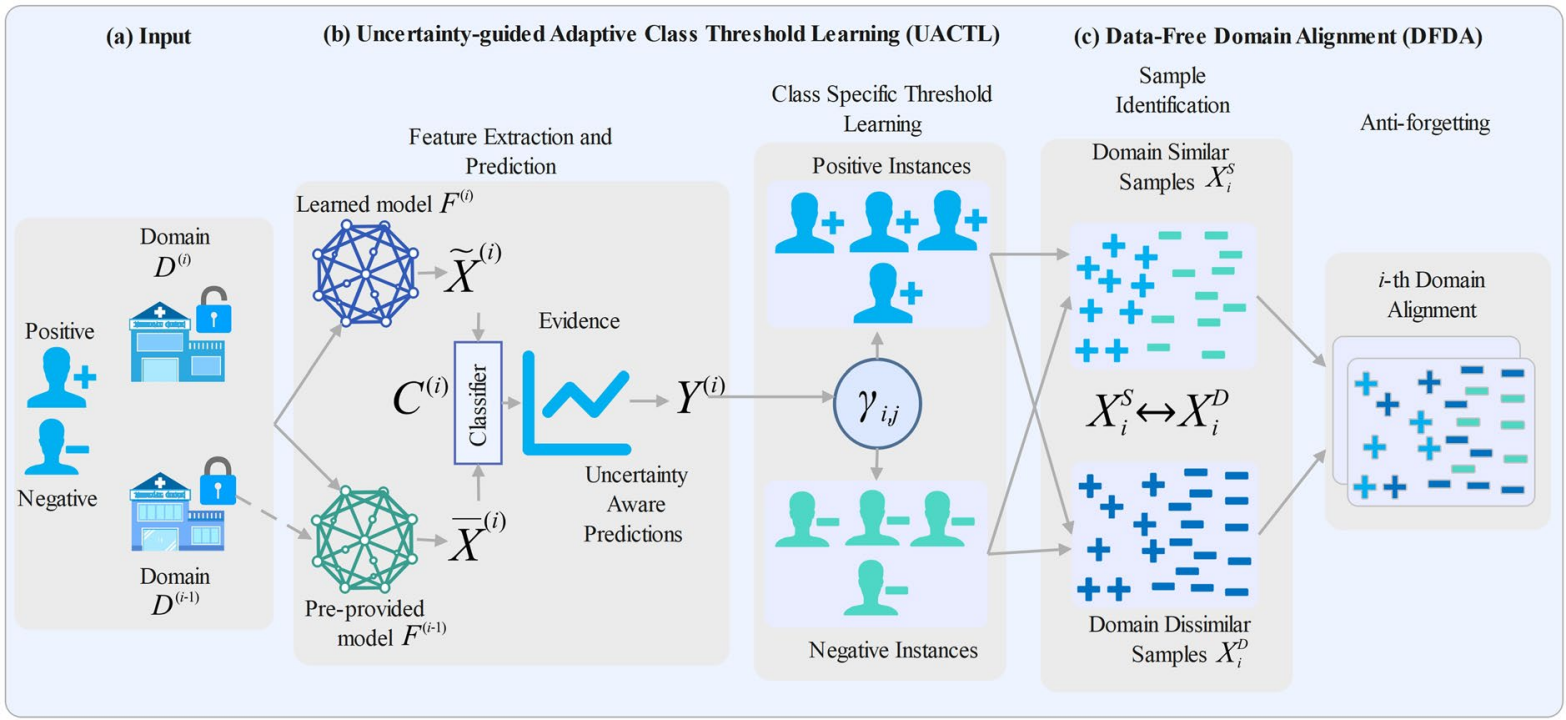
Overview of the proposed incremental learning framework for cross-domain depression disorder detection. The framework consists of three main components: (**a**) **Input**—embeddings are obtained from the current domain’s feature extractor $F^{(i)}$, while the previous extractor $F^{(i-1)}$ is reused to generate historical features; (**b**) **UACTL**—Uncertainty-guided Adaptive Class-Specific Threshold Learning dynamically computes class-specific thresholds using evidential uncertainty and divergence $\gamma _{i,j}$; (**c**) **DFDA**—Data-Free Domain Alignment module partitions data into domain-similar $\mathscr {X}^S_i$ and domain-dissimilar $\mathscr {X}^D_i$ sets and aligns them using MMD loss. This pipeline enables sequential adaptation without accessing historical data and mitigates catastrophic forgetting.

As illustrated in Fig. 1, our incremental learning framework comprises three main components: (a) Input, (b) Uncertainty-guided Adaptive Class-Specific Threshold Learning (UACTL), and (c) Data-Free Domain Alignment (DFDA). In the Input stage, due to privacy constraints, we access only the well-trained feature extractor $F^{(i-1)}$ from the previous domain, while the current domain $D^{(i)} = \{(X^{(i)}, Y^{(i)})\}$ provides the raw data $X^{(i)}$. The feature extractor $F^{(i)}$ produces embeddings $\tilde{X}^{(i)} = F^{(i)}(X^{(i)})$ for the current domain, and historical features are obtained as $\bar{X}^{(i)} = F^{(i-1)}(X^{(i)})$. The classifier $C^{(i)}$ outputs evidential predictions, deriving an evidence vector $e$ that is converted into Dirichlet parameters $\varvec{\alpha } = e + 1$, and yielding uncertainty-aware predictions $\tilde{Y}^{(i)} = C^{(i)}(\tilde{X}^{(i)})$ and $\bar{Y}^{(i)} = C^{(i)}(\bar{X}^{(i)})$. In the UACTL module, we compute the divergence $\gamma _{i,j}$ between the uncertainty-aware predictions using Jensen–Shannon divergence. We then define adaptive class-specific thresholds $\tau = \{\tau _p, \tau _n\}$, corresponding to the positive class ($m$) and the negative class ($h$), respectively, which robustly address class imbalance and filter out uncertain predictions. Finally, in the DFDA module, samples are partitioned into domain-similar sets $\mathscr {X}^S_{i}$ (i.e., samples with $\gamma _{i,j} < \tau$) and domain-dissimilar sets $\mathscr {X}^D_{i}$ (samples with $\gamma _{i,j} \ge \tau$), with each set further divided by class. An MMD loss is then applied to align the feature distributions of $\mathscr {X}^S_{i}$ and $\mathscr {X}^D_{i}$, thereby approximating the historical feature space and mitigating catastrophic forgetting. This iterative process across domains ensures the model retains its competence on previous domains while effectively adapting to new ones.

#### Uncertainty-guided adaptive class-specific threshold learning (UACTL)

This section introduces the UACTL module of our incremental learning framework for detecting depression disorder. In contrast to conventional methods that depend on predetermined, manually set thresholds based on prior knowledge and extensive experimentation, our approach dynamically learns class-specific thresholds to adapt to changing data distributions, building on insights from prior studies^7,53,54^. Due to the inherent class imbalance in clinical depression datasets, where minority MDD samples may be under-represented, we incorporate uncertainty into the thresholding process. We calculate the divergence between current and historical predictions using the Jensen–Shannon divergence and integrate uncertainty measures based on evidence scores and Dirichlet concentration parameters to enhance the thresholds. This approach, which accounts for uncertainty, guarantees that only samples with dependable, low-uncertainty predictions affect future domain alignment, thus improving model robustness and adaptability across various domains.

- *Feature Extraction and Prediction:* For each domain $D^{(i)} = \{(X^{(i)}, Y^{(i)})\}$, the framework initially extracts robust features utilising a pre-trained mBERT model. Given the raw data $X^{(i)}$ from the $i$th domain, the feature extractor $F^{(i)}$ generates contextualised embeddings represented as $\tilde{X}^{(i)} = F^{(i)}(X^{(i)})$. The embeddings are then input into the classifier $C^{(i)}$ to produce predictions, resulting in
  1 $$\begin{aligned} \tilde{Y}^{(i)} = C^{(i)}(F^{(i)}(X^{(i)})). \end{aligned}$$
  In this context, $C^{(i)}$ represents the classification network, and the classification loss associated with domain $i$ is defined as the standard cross-entropy loss:
  2 $$\begin{aligned} L^c_i = L_{\text {CE}}(C^{(i)}(F^{(i)}(X^{(i)})), Y^{(i)}), \end{aligned}$$
  where $Y^{(i)}$ denotes the ground-truth labels. To maintain conciseness, we denote the output $\tilde{Y}^{(i)}$ as the prediction, which is subsequently divided into positive and negative components based on the ground-truth labels, specifically, $\tilde{Y}^{(i)} = \{ \tilde{Y}^{(i,m)}, \tilde{Y}^{(i,h)} \}$. Due to privacy limitations and the lack of access to raw historical data $D^{(i-1)}$, we utilise the previously established feature extractor $F^{(i-1)}$ to obtain historical insights. Historical predictions are computed as:
  3 $$\begin{aligned} \bar{Y}^{(i)} = C^{(i)}(F^{(i-1)}(X^{(i)})), \end{aligned}$$
  where the embeddings $F^{(i-1)}(X^{(i)})$, referred to as $\bar{X}^{(i)}$, are derived by applying the preceding model $F^{(i-1)}$ to the current input $X^{(i)}$. The resulting historical predictions $\bar{Y}^{(i)}$ are also categorised into classes: $\bar{Y}^{(i)} = \{ \bar{Y}^{(i,m)}, \bar{Y}^{(i,h)} \}$, serving as a reference for the adaptive threshold learning module. At this stage, evidential uncertainty is incorporated by modifying the classifier $C^{(i)}$ to output evidence scores. These scores are used to compute Dirichlet concentration parameters, enabling a principled estimation of predictive uncertainty. This uncertainty estimation plays a key role in the adaptive thresholding process, ensuring that only reliable, low-uncertainty predictions are included in downstream domain alignment.

- *Uncertainty-guided Optimisation with Evidential Deep Learning (EDL):* To obtain robust uncertainty-aware predictions, we adopt EDL. EDL models the output as parameters of a Dirichlet distribution rather than a single point estimate, precisely quantifying uncertainties, thereby deriving accurate recognition results. This is crucial in the screening for clinical depression, where patients’ responses can be noisy, ambiguous, and distributional changes are common. In this case, predictive confidence plays a critical role in safe decision-making and improves interpretability. Unlike traditional classifiers that provide class probabilities through a softmax layer, we modify the classifier $C^{(i)}$ to generate evidence vectors for the purpose of uncertainty quantification. For an input instance $X_{i,j}$, the classifier produces an evidence vector $e_{i,j} \in \mathbb {R}_{+}^K$, where $K = 2$, representing the *m* and *h* classes. The evidence values are non-negative and indicate the support for each class. The evidence vector is transformed into parameters of a Dirichlet distribution, which represents the probability density across class assignments. This formulation enables the model to represent varying degrees of confidence, with high total evidence yielding confident predictions and low evidence resulting in flat, uncertain predictions. Formally, this is presented as:
  4 $$\begin{aligned} \alpha _{i,j} = e_{i,j} + 1 \end{aligned}$$
  where $\alpha _{i,j} = [\alpha _{i,j}^{(1)}, \alpha _{i,j}^{(2)}]$ denotes the Dirichlet parameters for instance $X_{i,j}$. The expected class probabilities are obtained from the Dirichlet distribution as follows:
  5 $$\begin{aligned} \hat{p}_{i,j}^{(k)} = \frac{\alpha _{i,j}^{(k)}}{S_{i,j}}, \quad \text {where} \quad S_{i,j} = \sum _{k=1}^{K} \alpha _{i,j}^{(k)} \end{aligned}$$
  where $S_{i,j}$ denotes the comprehensive evidence for $X_{i,j}$. We calculate the model’s uncertainty as follows:
  6 $$\begin{aligned} u_{i,j} = \frac{K}{S_{i,j}} \end{aligned}$$
  where $u_{i,j} \in [0, 1]$. Lower total evidence ($S_{i,j}$) indicates greater uncertainty ($u_{i,j}$), which reflects the model’s confidence in its predictions. To train the model for improved and well-calibrated evidence and uncertainty, we substitute the standard cross-entropy loss with an evidential loss $\mathscr {L}_{\text {EDL}}$, which penalises both misclassification and overconfidence. For an instance $X_{i,j}$ with ground-truth label $Y_{i,j}$, we compute the Adaptive Evidential Cross-Entropy Loss ($\mathscr {L}_{\text {ACE}}$) by calculating the expected log-likelihood according to the Dirichlet distribution as follows:
  7 $$\begin{aligned} \mathscr {L}_{\text {ACE}}(\alpha _{i,j}) = \sum _{k=1}^{K} Y_{i,j}^{(k)} \left[ \psi (S_{i,j}) - \psi (\alpha _{i,j}^{(k)}) \right] \end{aligned}$$
  where $\psi (\cdot )$ denotes the digamma function. This loss term correlates the predicted evidence with the actual labels, while explicitly considering the model’s uncertainty. The goal of the adaptive loss function is to adjust the model’s output parameters such that high confidence predictions are encouraged when sufficient evidence is available, while allowing the model to maintain a degree of uncertainty when evidence is scarce. However, the adaptive loss function does not adequately address the problem of insufficient evidence resulting from incorrect labels. To mitigate the risk of the model attributing excessively high evidence to incorrect predictions, we apply Kullback-Leibler Divergence ($\mathscr {L}_{\text {KL}}$) regularisation to the Dirichlet parameters, guiding them towards a uniform distribution that signifies maximal uncertainty. This penalises the model when it becomes overconfident on uncertain or mislabeled instances, encouraging cautious learning behaviour during early training. The KL divergence loss is given by:
  8 $$\begin{aligned} \mathscr {L}_{\text {KL}}(\alpha _{i,j}) = \log \left( \frac{\Gamma (S_{i,j})}{\Gamma (K)} \right) - \sum _{k=1}^{K} (\alpha _{i,j}^{(k)} - 1) \left[ \psi (\alpha _{i,j}^{(k)}) - \psi (S_{i,j}) \right] \end{aligned}$$
  where $\Gamma (\cdot )$ denotes the gamma function. This regularisation term penalises deviations from the uniform Dirichlet prior $D(1)$. The final EDL loss function integrates $\mathscr {L}_{\text {ACE}}$ and $\mathscr {L}_{\text {KL}}$, incorporating an annealing coefficient $\lambda _t$ to gradually increase the impact of $\mathscr {L}_{\text {KL}}$:
  9 $$\begin{aligned} \mathscr {L}_{\text {EDL}} = \mathscr {L}_{\text {ACE}}(\alpha _{i,j}) + \lambda _t \cdot \mathscr {L}_{\text {KL}}(\alpha _{i,j}) \end{aligned}$$
  where $\lambda _t = \min \left( 1.0, \frac{t}{E} \right)$. Here, $t$ represents the current training epoch, and $E$ denotes the total number of annealing epochs. The annealing mechanism mitigates the risk of early convergence to high certainty on incorrectly labelled samples, ensuring a gradual and stable optimisation process.

- *Class-Specific Threshold Learning:* In our framework, the class-specific threshold learning strategy is designed to dynamically learn the model’s decision boundaries for each class by comparing the evidential predictions from the current feature extractor $F^{(i)}$ and the previous extractor $F^{(i-1)}$. Specifically, let $\tilde{Y}^{(i)}$ denote the class probability distribution predicted by $F^{(i)}$ (derived from the Dirichlet parameters computed via our evidential output layer), and $\bar{Y}^{(i)}$ denote the distribution predicted by $F^{(i-1)}$. To quantify the discrepancy between these distributions within each class, we compute the Jensen–Shannon (JS) divergence. We choose JS divergence because it is symmetric, bounded, and more stable than other divergence measures when handling subtle shifts in probability distributions. The JS divergence is defined as the mean of the Kullback–Leibler (KL) divergence between the two distributions in both directions:
  10 $$\begin{aligned} \begin{aligned} \gamma _{i}^{(m)}&= \frac{1}{2} \left( D_{\text {KL}} \left( \tilde{Y}^{(i,m)} \,\Vert \, \bar{Y}^{(i,m)} \right) + D_{\text {KL}} \left( \bar{Y}^{(i,m)} \,\Vert \, \tilde{Y}^{(i,m)} \right) \right) \\ \gamma _{i}^{(h)}&= \frac{1}{2} \left( D_{\text {KL}} \left( \tilde{Y}^{(i,h)} \,\Vert \, \bar{Y}^{(i,h)} \right) + D_{\text {KL}} \left( \bar{Y}^{(i,h)} \,\Vert \, \tilde{Y}^{(i,h)} \right) \right) \end{aligned} \end{aligned}$$
  where $D_{\text {KL}}(\cdot \,\Vert \, \cdot )$ denotes the Kullback–Leibler (KL) divergence. Here, $\tilde{Y}^{(i,m)}$ and $\tilde{Y}^{(i,h)}$ represent the probability distributions for the *m* and *h* classes, respectively. The JS divergences $\gamma _{i}^{(m)}$ and $\gamma _{i}^{(h)}$ measure how the predictions of $F^{(i)}$ deviate from $F^{(i-1)}$ while accounting for uncertainty encoded in their evidential outputs. This joint consideration allows the model not only to quantify distributional shifts, but also to assess prediction reliability, providing a principled basis for class-specific threshold estimation. For instance, the model can discount low-confidence predictions even if their divergences are small, thereby avoiding false positives from unreliable outputs. This dual criterion ensures that only predictions which are both semantically consistent (low JS divergence) and statistically reliable (low uncertainty) inform the thresholding process. The learned thresholds for each class are then computed by combining the statistical properties of the divergence measures. Specifically, we define the thresholds as:
  11 $$\begin{aligned} \begin{aligned} \tau _{i}^{(m)}&= \mu (\gamma _{i}^{(m)}) - \alpha _{i}^{(m)} \cdot \sigma (\gamma _{i}^{(m)}) \\ \tau _{i}^{(h)}&= \mu (\gamma _{i}^{(h)}) - \alpha _{i}^{(h)} \cdot \sigma (\gamma _{i}^{(h)}) \end{aligned} \end{aligned}$$
  where $\mu (\cdot )$ and $\sigma (\cdot )$ denote the mean and standard deviation of the divergence values, and $\alpha _{i}^{(m)}$, $\alpha _{i}^{(h)}$ are class-specific hyperparameters controlling threshold sensitivity. This process allows the model to adjust its threshold in response to the changing distribution of each class, thereby improving performance across different datasets and domains. This approach develops a precise and efficient sample selection strategy to identify samples that are similar in domain, particularly in dynamic data scenarios marked by class imbalance. The class-specific threshold refinement mechanism promotes iterative threshold adjustment for each class throughout the training process. Furthermore, customising thresholds to correspond with the distinct attributes of each class significantly mitigates the likelihood of under-representing or misclassifying minority classes. Consequently, the model exhibits increased versatility in handling diverse data patterns, thereby improving its adaptability and robustness across different domains.

#### Data-free domain alignment (DFDA)

In practical scenarios, it is essential for models to adjust to new datasets characterised by different feature distributions, all while preserving the knowledge acquired from prior domains. Consequently, models often lose previously acquired knowledge when adjusting to a new domain, a phenomenon referred to as *catastrophic forgetting*. In response to this challenge, we present our *Data-Free Domain Alignment* (DFDA) module. Due to limitations imposed by privacy and the lack of access to raw historical data, DFDA utilises the existing domain data to estimate the feature distribution of earlier domains. The DFDA module initially divides the current domain $D^{(i)}$ into two sample sets: domain-similar ($\mathscr {X}^S_i$) and domain-dissimilar ($\mathscr {X}^D_i$). This partitioning is informed by the adaptive thresholds established in the UACTL module. We then implement alignment constraints through the *Maximum Mean Discrepancy* (MMD) operator to reduce the distributional disparity between these sets. This method successfully captures historical feature patterns and reduces the risk of catastrophic forgetting, all without needing direct access to previous raw data, as shown in related studies.

- *Sample Identification:* Our framework utilises a class-tailored sample identification strategy to effectively capture historical information without the need for raw data access, relying on the UACTL module. For each domain $D^{(i)}$, we calculate the divergence $\gamma _{i,j}^{(k)}$ between the current prediction $\tilde{Y}_{i,j}^{(k)}$ and the historical prediction $\bar{Y}_{i,j}^{(k)}$ for each class $k \in \{ m, h \}$. Utilising the established class-specific thresholds $\tau _i^{(k)}$ (refer to Eq. (11)), samples are categorised into sets that are either domain-similar or domain-dissimilar. Accordingly, the sample sets are partitioned as follows:
  12 $$\begin{aligned} \begin{aligned} \mathscr {X}_i^{S,(m)}&= \left\{ X_{i,j}^{(m)} \,\Bigg |\, \gamma _{i,j}^{(m)}< \tau _i^{(m)}, \, j = 1, \dots , Q_i^{(m)} \right\} \\ \mathscr {X}_i^{D,(m)}&= \left\{ X_{i,j}^{(m)} \,\Bigg |\, \gamma _{i,j}^{(m)} \ge \tau _i^{(m)}, \, j = 1, \dots , Q_i^{(m)} \right\} \\ \mathscr {X}_i^{S,(h)}&= \left\{ X_{i,j}^{(h)} \,\Bigg |\, \gamma _{i,j}^{(h)} < \tau _i^{(h)}, \, j = 1, \dots , Q_i^{(h)} \right\} \\ \mathscr {X}_i^{D,(h)}&= \left\{ X_{i,j}^{(h)} \,\Bigg |\, \gamma _{i,j}^{(h)} \ge \tau _i^{(h)}, \, j = 1, \dots , Q_i^{(h)} \right\} \end{aligned} \end{aligned}$$
  where $Q_i^{(m)}$ and $Q_i^{(h)}$ denote the number of positive and negative samples, respectively. The union of the domain-similar sets forms:
  13 $$\begin{aligned} \mathscr {X}_i^S = \mathscr {X}_i^{S,(m)} \cup \mathscr {X}_i^{S,(h)} \end{aligned}$$
  and similarly, the domain-dissimilar set is given by:
  14 $$\begin{aligned} \mathscr {X}_i^D = \mathscr {X}_i^{D,(m)} \cup \mathscr {X}_i^{D,(h)} \end{aligned}$$
  The union operator $\cup$ integrates the samples from both classes. This sophisticated sample identification strategy, based on adaptive thresholds and evidential uncertainty, allows our model to strategically align dependable, low-uncertainty samples, thus improving domain alignment and reducing the risk of catastrophic forgetting.

- *Mitigating Catastrophic Forgetting:* Traditional methods for domain incremental learning frequently depend on the replay of past samples or the generation of new ones to maintain previously learnt knowledge and reduce the risk of catastrophic forgetting. Nevertheless, privacy constraints within our application impose significant limitations on access to raw historical data. In response to this challenge, we propose a domain alignment mechanism that operates without data, utilising the domain-similar sample set $\mathscr {X}^S_i$ (as identified in Eq. (13)) to effectively approximate the feature distribution of the prior domain. We align $\mathscr {X}^S_i$ with the domain-dissimilar sample set $\mathscr {X}^D_i$ calculated using MMD as follows:
  15 $$\begin{aligned} \mathscr {L}^{(i)}_D = \text {MMD}(\mathscr {X}^S_i, \mathscr {X}^D_i), \end{aligned}$$
  where, $\mathscr {L}^{(i)}_D$ is the MMD loss and $\text {MMD}(\cdot , \cdot )$ represents the squared Maximum Mean Discrepancy (MMD$^2$) operator , defined as
  16 $$\begin{aligned} \text {MMD}^2(\mathscr {X}_i^S, \mathscr {X}_i^D) = \frac{1}{|\mathscr {X}_i^S|^2} \sum _{X, X' \in \mathscr {X}_i^S} \kappa (X, X') + \frac{1}{|\mathscr {X}_i^D|^2} \sum _{Z, Z' \in \mathscr {X}_i^D} \kappa (Z, Z') - \frac{2}{|\mathscr {X}_i^S||\mathscr {X}_i^D|} \sum _{X \in \mathscr {X}_i^S} \sum _{Z \in \mathscr {X}_i^D} \kappa (X, Z), \end{aligned}$$
  where $X, X', Z, Z'$ are individual feature vectors drawn from the sets $\mathscr {X}_i^S$ and $\mathscr {X}_i^D$, respectively, and $\kappa (\cdot , \cdot )$ denotes the Gaussian Radial Basis Function (RBF) kernel defined as
  17 $$\begin{aligned} \kappa (X, Z) = \exp \left( -\frac{\Vert X - Z\Vert ^2}{2\sigma ^2}\right) . \end{aligned}$$
  where $\sigma$ is the kernel bandwidth hyperparameter. The alignment strategy is grounded in the assumption that the domain-similar samples $\mathscr {X}^S_i$, identified via predictive agreement with the prior domain’s feature extractor $F^{(i-1)}$, capture semantic and statistical characteristics consistent with the historical domain $D^{(i-1)}$. As a result, aligning $\mathscr {X}^S_i$ with the domain-dissimilar set $\mathscr {X}^D_i$ acts as a surrogate for prior domain alignment, enabling the model to preserve previously learned domain-invariant features without needing access to original data. This effectively reduces the disparity between the distributions of historical and incoming data and has been shown to effectively mitigate forgetting  ^7^.

#### Loss function

The overall loss function is structured to fulfil two main objectives: (i) to accurately classify data from the current domain using uncertainty-aware predictions and (ii) to align the feature distribution of the current domain with historical patterns in order to reduce the risk of catastrophic forgetting. In the initial domain ($i = 1$), the training process utilises the evidential loss function defined as:

18 $$\begin{aligned} \mathscr {L}^{(1)}_{\text {EDL}} = \mathscr {L}_{\text {ACE}}(\varvec{\alpha }^{(1)}) + \lambda _t\, \mathscr {L}_{\text {KL}}(\varvec{\alpha }^{(1)}) \end{aligned}$$

where, $\mathscr {L}^{(1)}_{\text {EDL}}$ denotes the evidential loss for the initial domain $D^{(1)}$, $\mathscr {L}_{\text {ACE}}$ is the adaptive cross-entropy loss, and $\mathscr {L}_{\text {KL}}$ is the KL divergence regularisation term computed from the Dirichlet parameters $\varvec{\alpha }^{(1)}$. For subsequent domains ($n \ge 2$), the total loss function is articulated as follows:

19 $$\begin{aligned} \mathscr {L}_{\text {total}} = \mathscr {L}^{(1)}_{\text {EDL}} + \sum _{i=2}^{n} \Big ( \mathscr {L}^{(i)}_{\text {EDL}} + \lambda \, \mathscr {L}^{(i)}_D \Big ) \end{aligned}$$

where $\mathscr {L}^{(i)}_D$ is the and $\lambda$ serves as a hyper-parameter that regulates the trade-off between the acquisition of new domain-specific features and the retention of historical knowledge. When $\lambda$ is small, the model emphasises current classification performance; as $\lambda$ increases, the model progressively prioritises the alignment of feature distributions to preserve prior knowledge. This unified loss function ensures that the model adapts to new domains while retaining competence in previously learnt tasks.

## Experiments

In this section, we first introduce the experiment setup and comparison methods.

### Experiment setting

We use a pre-trained mBERT model to extract sentence-level features from transcripts, which are then fed into our classifier that produces evidence vectors converted into Dirichlet parameters. The model is trained sequentially over multiple domains, and we evaluate its performance using the metrics accuracy (ACC), recall (REC), specificity (SPE), precision (PRE), F1-score (F1), area under the receiver operating Characteristic curve (AUROC) and area under the precision-recall curve (AUPR). Further we report Macro-F1 score and Balanced Accurracy (Bal-ACC) in the suplementary material.

#### Datasets

We evaluate our proposed framework on four benchmark datasets. The datasets were obtained with informed consent following ethical standards for research involving human participants. In our experiment, we focus exclusively on transcripts.

- *Chinese Multimodal Depression Corpus (CMDC):* Chinese Multimodal Depression Corpus^6^ is a dataset designed to support Major Depressive Disorder (MDD) assessment in China. The dataset comprises 78 participants (26 diagnosed with MDD, 52 healthy controls) responding to 12 standardized interview questions. Each interview includes text transcripts and audio-visual recordings, with depression severity labeled using the Patient Health Questionnaire-9 (PHQ-9). A small subset of responses (three participants) for the 10th interview question were not recorded. Since experiments for each question are conducted independently, we exclude missing samples only for the affected question. All participants provided written informed consent, and the study was approved by the Independent Medical Ethics Committee Board of Beijing Anding Hospital (Application 2019 No. 53) in accordance with the Declaration of Helsinki.
- *DAIC-WoZ Dataset:* The DAIC-WoZ corpus^55^ contains 189 clinical interviews conducted via a virtual interviewer (Ellie) using a “Wizard of Oz” paradigm. Participants’ depression status is determined by PHQ-8 scores, with binary labels (depressed/non-depressed) derived from a threshold of $\ge$10. The dataset is partitioned into training (107 participants), development (35), and test (47) sets. Class distribution is imbalanced with 56 depressed and 133 non-depressed participants. The original data collection was conducted under ethical guidelines by the University of Southern California, with informed consent obtained from all participants as part of the project’s institutional review process.
- *Emotional Audio-Textual Depression Corpus (EATD):* The Emotional Audio-Textual Depression Corpus^56^ is a dataset focused on Major Depressive Disorder (MDD) in Chinese individuals, developed by Tongji University. It includes interview recordings and textual transcripts from 162 recruited student volunteers, along with their respective SDS scores. An SDS score of 53 or higher indicates the presence of Major Depressive Disorder (MDD). Similar to the setup in^5^, we exclude samples with excessively short speech durations from the dataset, resulting in 19 MDD samples and 83 NC samples. This dataset is publicly available and was used in accordance with the terms set by the original authors.
- *Multimodal Open Dataset for Mental-disorder Analysis (MODMA):* The Multimodal Open Dataset for Mental-disorder Analysis^57^ is developed by Lanzhou University and comprises audio and EEG data from clinically diagnosed Major Depressive Disorder (MDD) and Normal Controls (NC). Subjects with MDD are recruited at the Second Hospital of Lanzhou University, a premier institution in Gansu, China, while the poster targets the non-clinical (NC) population. The audio signal utilised in this experiment involved 52 subjects, comprising 28 in the non-clinical group and 24 with MDD, engaged in interviews, readings, and picture descriptions. Additionally, we employ online speech transcription software to convert audio signals into text, followed by manual inspections. Written informed consent was obtained from all participants. The study design and consent procedures were approved by the Ethics Committee for Biomedical Research at the Second Hospital of Lanzhou University in accordance with the Declaration of Helsinki.

#### Experiment design

In the design of our experiment, each dataset is considered as a separate domain. For instance, in *Task #1* (CMDC $\rightarrow$ DAIC-WoZ $\rightarrow$ EATD $\rightarrow$ MODMA), the model undergoes an initial training phase utilising the CMDC dataset (Domain #1). Next, it is fine-tuned on the DAIC-WoZ dataset (Domain #2), followed by further fine-tuning on the EATD dataset (Domain #3). The process is repeated on Domain #4 (MODMA dataset), resulting in the final model. The trained network is then applied to the test sets of all four datasets for performance evaluation. This approach allows us to assess the effectiveness of our method in adapting to the new task while maintaining previously acquired knowledge.

#### Comparison methods

We assess the efficacy of our framework by comparing it with baseline and domain-incremental learning (DIL) approaches, in addition to various domain adaptation (DA) methods similar to those proposed by Chen et al.^7^.

1. **Baseline methods**
  *Baseline1 (B1):* This merges all datasets into a single composite dataset by combining their respective training, validation, and testing partitions into unified sets. It provides a bottom bound reference against which incremental learning techniques can be evaluated. Even though the model is trained on the combined data, we still report performance individually per dataset to assess how well this broad training generalizes.
  *Baseline2 (B2):* The Baseline2 first trains a model on Domain #1, then fine-tunes its parameters on Domain #2. In relation to the CMDC $\rightarrow$ DAIC-WOZ task, we first train a model using the CMDC dataset and subsequently fine-tune its parameters on the DAIC-WOZ dataset. We then apply the well-trained network to the test set, specifically the test data from DAIC-WOZ, to obtain the final prediction results.
2. **DIL-based comparison methods**
  *DIL-MDD *^7^: Serves as a domain-incremental baseline specifically designed for MDD detection. It sequentially adapts a single model across multiple domains, focusing on mitigating catastrophic forgetting by leveraging previous knowledge while being fine-tuned on new data.
  *Replay-based Method (RM-based):* The method retains high-confidence samples, determined by a learnt threshold, from prior domains and integrates them with data from the current domain for training purposes. Samples that do not surpass this threshold are eliminated, which may worsen class imbalance while facilitating the retention of prior knowledge.
  *Generative-based Method (GM-based):* It statistically synthesizes virtual samples for previous domains by approximating their feature distributions (using estimated means and standard deviations). The model is then trained jointly on both real and synthetic data to maintain knowledge of earlier domains.
3. **DA-based methods** While DA methods generally prioritise adaptation to a target domain over the retention of knowledge from the source domain, we broaden their evaluation to encompass both new and existing domains for a comprehensive comparison.
  *MiniMax Entropy (MME)*^58^: A method that mitigates domain discrepancy through the learning of representative prototypes and the classification of features based on similarity.
  *DANN*^59^: DANN is designed to learn discriminative and domain-invariant features via a joint optimisation which entails the optimisation of the underlying features alongside two discriminative classifiers that function on these features.
  *DALN*^60^: This method provides explicit domain alignment and class differentiation through the use of predicted discriminative information.

We re-implement the code provided by the authors of the mentioned papers and conduct a thorough comparison. For DIL-MDD, we re-implement it according to the descriptions provided in the paper. To ensure a fair comparison, we adapt the method to fit our experimental design while preserving its core design principles.

## Results and discussion

### Results analysis

In this section, we thoroughly evaluate the proposed method against other approaches across multiple classification tasks. Key metrics are reported per domain following sequential training. We also include ablation studies to assess the effect of uncertainty-guided thresholding and hyperparameter tuning.

#### Performance evaluation

In the following, we comprehensively evaluate the proposed method, UDIL-DD, against comparison baselines across four incremental MDD classification tasks. The detailed results are presented in Table 1. To further assess performance under class imbalance, we report Macro-F1 and Balanced Accuracy scores in Table S1 of the Supplementary Material.

**Table 1:** Performance comparison across different MDD tasks using various DIL methods. Each subtable corresponds to a unique task sequence.

**Method**	CMDC					DAIC-WoZ					EATD					MODMA				
(a) Task #1: CMDC $\rightarrow$ DAIC-WoZ $\rightarrow$ EATD $\rightarrow$ MODMA																				
	ACC	PRE	REC	SPE	F1	ACC	PRE	REC	SPE	F1	ACC	PRE	REC	SPE	F1	ACC	PRE	REC	SPE	F1
Baseline1	52.85	29.41	33.25	57.14	31.21	55.42	57.98	39.31	67.86	46.85	65.36	31.25	45.83	71.18	37.16	72.40	32.98	33.76	77.20	33.37
RM-based	65.67	47.98	50.63	64.29	49.27	70.80	**70.93**	54.41	**75.00**	61.58	66.02	32.14	36.83	73.08	34.33	**81.78**	34.26	10.67	93.87	16.27
GM-based	68.24	50.84	50.67	71.43	50.75	63.08	59.31	54.45	64.29	56.78	67.31	32.41	36.15	**73.43**	34.18	81.75	40.98	22.59	**96.15**	29.13
DIL-MDD	70.80	54.65	61.68	71.46	57.95	70.80	67.65	64.71	71.46	66.15	69.23	34.78	54.17	71.43	42.36	70.32	33.12	**48.04**	73.11	39.21
UDIL-DD	**73.26**	**59.30**	**68.71**	**72.21**	**63.66**	**76.19**	67.65	**69.33**	72.21	**68.48**	**70.51**	**35.90**	**55.56**	72.36	**43.62**	71.70	**52.94**	48.04	73.14	**50.37**

**Method**	DAIC-WoZ					CMDC					MODMA					EATD				
(b) Task #2: DAIC-WoZ $\rightarrow$ CMDC $\rightarrow$ MODMA $\rightarrow$ EATD																				
	ACC	PRE	REC	SPE	F1	ACC	PRE	REC	SPE	F1	ACC	PRE	REC	SPE	F1	ACC	PRE	REC	SPE	F1
Baseline1	70.91	23.97	28.89	81.67	26.20	52.85	20.40	28.38	61.61	23.74	55.42	49.97	34.44	74.33	40.78	57.36	44.83	33.50	**72.44**	38.35
RM-based	68.20	25.91	37.19	74.16	30.54	57.31	33.31	43.75	64.08	37.82	52.19	49.98	57.60	49.80	53.52	58.04	46.67	39.29	68.57	42.66
GM-based	68.76	33.63	51.31	74.18	40.63	64.11	39.95	46.76	72.53	43.09	54.86	49.98	42.98	65.39	46.22	63.27	44.62	41.67	67.37	43.09
DIL-MDD	74.36	35.07	80.87	81.97	48.92	67.07	43.15	52.09	72.63	47.20	65.78	**61.83**	64.21	65.49	63.00	66.08	66.39	65.45	66.01	65.92
UDIL-DD	**75.19**	**36.36**	**82.56**	**82.05**	**50.49**	**67.90**	**43.68**	**56.25**	**73.01**	**49.17**	**66.71**	61.68	**65.76**	**66.01**	**63.65**	**67.13**	**66.68**	**66.03**	66.01	**66.35**

**Method**	EATD					MODMA					CMDC					DAIC-WoZ				
(c) Task #3: EATD $\rightarrow$ MODMA $\rightarrow$ CMDC $\rightarrow$ DAIC-WoZ																				
	ACC	PRE	REC	SPE	F1	ACC	PRE	REC	SPE	F1	ACC	PRE	REC	SPE	F1	ACC	PRE	REC	SPE	F1
Baseline1	51.25	40.26	35.83	65.29	37.92	53.82	68.83	41.89	76.01	52.08	70.80	43.83	36.26	**80.27**	39.69	70.83	25.00	27.78	85.35	26.32
RM-based	52.97	53.44	56.83	68.67	55.08	58.10	**78.44**	60.58	**78.02**	68.36	67.18	51.77	27.81	84.36	36.18	81.86	25.93	18.89	**96.89**	21.86
GM-based	52.18	42.41	**58.15**	68.00	49.05	46.97	55.88	**61.30**	60.86	58.46	60.64	39.55	24.08	83.08	29.93	**83.08**	32.14	20.76	82.72	25.23
DIL-MDD	69.23	54.78	54.17	**72.73**	54.47	74.04	72.78	58.92	70.05	65.12	73.56	48.25	52.25	70.77	50.17	70.16	34.78	45.56	71.70	39.45
UDIL-DD	**70.51**	**55.90**	55.56	**72.73**	**55.73**	**74.52**	73.90	60.31	70.08	**66.42**	**73.96**	**62.75**	**53.63**	68.95	**57.83**	74.19	**46.66**	**48.33**	67.86	**47.48**

**Method**	MODMA					EATD					DAIC-WoZ					CMDC				
(d) Task #4: MODMA $\rightarrow$ EATD $\rightarrow$ DAIC-WoZ $\rightarrow$ CMDC																				
	ACC	PRE	REC	SPE	F1	ACC	PRE	REC	SPE	F1	ACC	PRE	REC	SPE	F1	ACC	PRE	REC	SPE	F1
Baseline1	54.16	54.44	35.33	71.18	42.85	71.14	25.00	29.78	**80.52**	27.18	51.59	10.33	29.27	60.46	15.27	52.85	10.33	25.45	**77.14**	14.70
RM-based	58.62	50.00	51.67	63.08	50.82	59.26	14.89	40.56	61.61	21.78	57.28	24.02	38.03	59.08	29.44	57.31	31.82	38.48	60.00	34.83
GM-based	58.64	54.46	51.67	72.23	53.03	63.45	20.78	**57.23**	68.75	30.49	62.49	34.61	**63.79**	**73.08**	44.87	62.97	32.12	50.00	72.86	39.11
DIL-MDD	65.38	61.54	54.44	72.31	57.77	71.55	41.84	60.00	71.39	49.30	**70.51**	54.40	58.99	72.31	56.60	69.24	45.48	54.55	65.71	49.60
UDIL-DD	**67.31**	**62.94**	**57.22**	**72.36**	**59.94**	**72.12**	**44.24**	58.56	71.31	**50.40**	71.07	**55.36**	59.51	72.19	**57.36**	**70.05**	**46.84**	**56.25**	67.86	**51.12**

Across all tasks, UDIL-DD consistently outperforms other methods across key metrics, including ACC, REC, and F1. In Task #1, UDIL-DD achieves the highest F1 scores on all four domains, with notable gains on CMDC (+5.71%) and DAIC-WoZ (+2.33%) compared to DIL-MDD. In Task #2, UDIL-DD maintains strong performance across domains, recording the highest ACC (75.19%) and F1 (50.49%) on DAIC-WoZ, and outperforming all methods on CMDC, MODMA, and EATD. Similar trends are observed in **Task #3**, where UDIL-DD exhibits improved balance across all metrics. The improvement in F1 compared to DIL-MDD is +1.26% on EATD, +1.3% on MODMA, and +7.38% on DAIC-WoZ. In Task #4, the proposed method demonstrates superior performance, attaining the highest F1 scores across all datasets, with recorded values of 59.94% on MODMA and 51.12% on CMDC. Although RM-based and GM-based methods demonstrate strong performance in ACC or SPE, they frequently exhibit reduced REC and F1 scores. For instance, in Task #1, the GM-based method attains a 96.15% specificity on MODMA, yet reports only 22.59% recall and 29.13% F1 score, highlighting deficiencies in minority class recognition and prediction bias. This prediction bias is also observed in Task #3 on the DIAC-WoZ domain. In contrast, UDIL-DD consistently attains high REC while maintaining specificity, demonstrating balanced and reliable predictions. From these observations, we can conclude that our proposed method improves overall classification performance and attains a more balanced performance across various metrics when applied to imbalanced datasets. The method effectively differentiates between minority and majority classes, thus reducing prediction biases identified in comparative approaches. This emphasises its robustness, effectiveness, and ability to scale in addressing the core challenges related to mental health detection tasks.

#### Performance comparison with domain adaptation methods

**Fig. 2:**
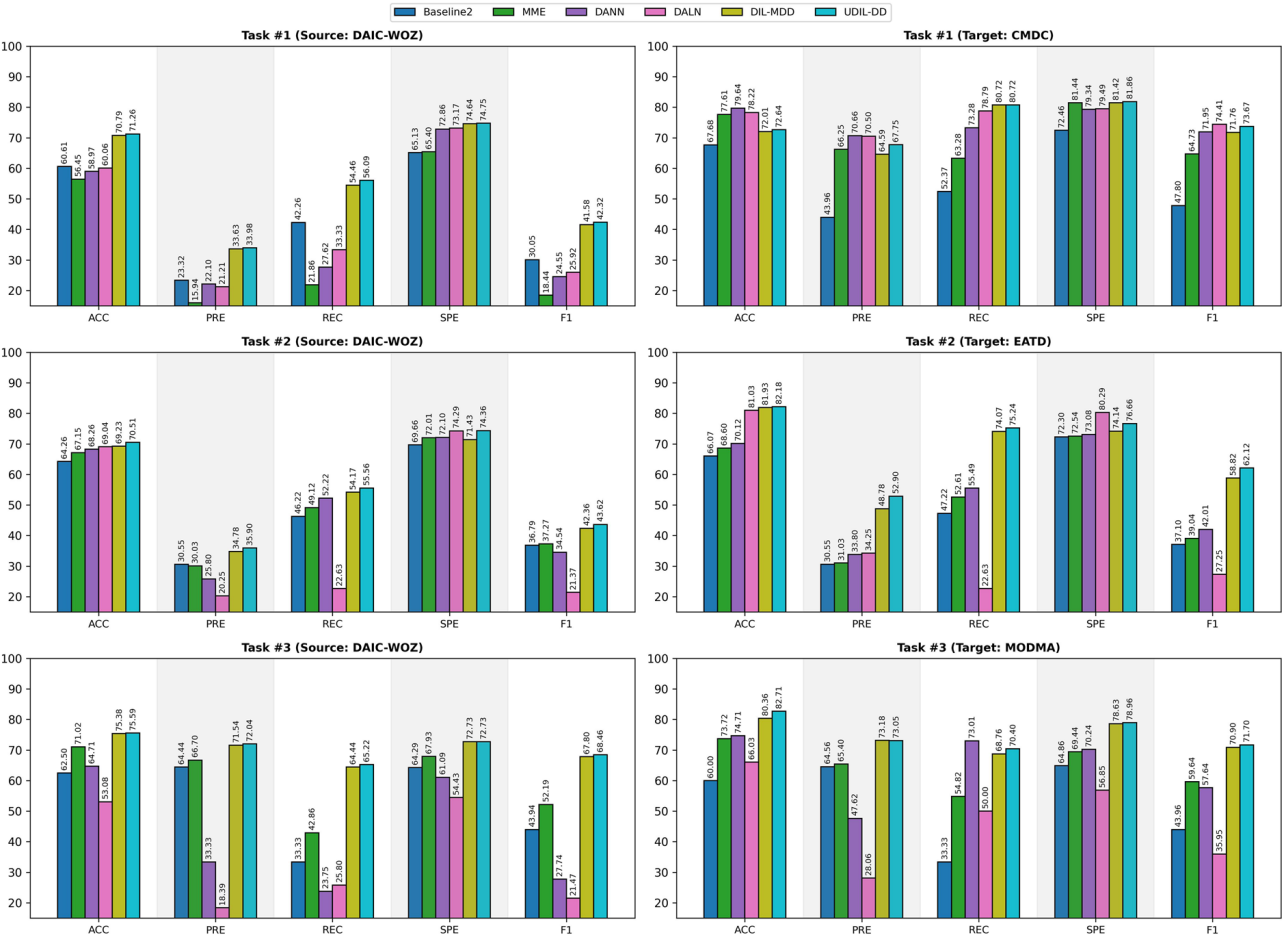
Comparative performance of domain adaptation (DA) and domain-incremental learning (DIL) methods across source and target domains.

Our approach is further compared with traditional domain adaptation (DA) techniques, specifically MME, DANN, and DALN. Domain adaptation methods focus on reducing feature discrepancies by aligning the source and target domains through explicit constraints and fine-tuning on target validation sets. In contrast, our setting represents a domain-incremental learning (DIL) scenario, where access to previous domain data is not available during subsequent training phases. Instead, the model is sequentially exposed to each domain, mirroring more realistic deployment conditions. To evaluate this, we design three tasks by varying the target domain while keeping the source domain fixed as DAIC-WoZ. Specifically, we report results for: (a) DAIC-WoZ $\rightarrow$ CMDC, (b) DAIC-WoZ $\rightarrow$ EATD, and (c) DAIC-WoZ $\rightarrow$ MODMA. As illustrated in Fig. 2, we present the comparative performance of all methods across source and target domains to assess both generalization and knowledge retention.

Across all tasks, DA methods generally demonstrate strong performance in the target domain; however, they show significant performance declines in the source domain. This indicates that they tend to overfit to new domains while compromising performance in previously learned domains. In Task #1, DALN demonstrates robust performance on CMDC with an F1 score of 74.41%, while its performance on DAIC-WoZ declines to 25.92%. Similarly, DANN and MME exhibit significant performance discrepancies across different domains. DIL-based approaches, particularly UDIL-DD, demonstrate consistently high performance across both domains. In Task #1, UDIL-DD records an F1 score of 42.32% on DAIC-WoZ and 73.67% on CMDC, surpassing DIL-MDD by 0.74% and 1.91%, respectively. In Task #2, DA methods similarly encounter challenges in achieving a balance in domain performance. For instance, DALN demonstrates an F1 score of 21.37% on DAIC-WoZ, indicating strong specificity. In contrast, UDIL-DD achieves an F1 score of 43.62%, which is 1.26% greater than that of DIL-MDD. On the target domain (EATD), UDIL-DD achieves the best F1 score (62.12%), outperforming DIL-MDD (58.82%) by a margin of 3.3%.

In Task #3, UDIL-DD surpasses DIL-MDD by 0.66% in F1 score on DAIC-WoZ (68.46% vs. 67.80%) and by 0.8% on MODMA (71.70% vs. 70.90%), while significantly outperforming all DA methods. From the results, DIL-based methods—DIL-MDD and UDIL-DD—demonstrate robust performance across both domains. Notably, UDIL-DD achieves higher and more competitive performance across all tasks. This indicates that our method achieves a balanced performance by effectively preserving source domain knowledge while adapting to new domains, a critical requirement in dynamic MDD detection scenarios. In particular, UDIL-DD, by leveraging its uncertainty awareness, effectively captures the inherent variability present in the data. This process allows for the retention of essential information from the source domain while seamlessly integrating new target domain features. The balanced performance highlights the robustness and generalisability of our approach in comparison to DIL-MDD and DA methods. Further domain adaptation results, where MODMA is used as the source domain, are presented in Figure S1 of the Supplementary Material.

#### Ablation study

**Fig. 3:**
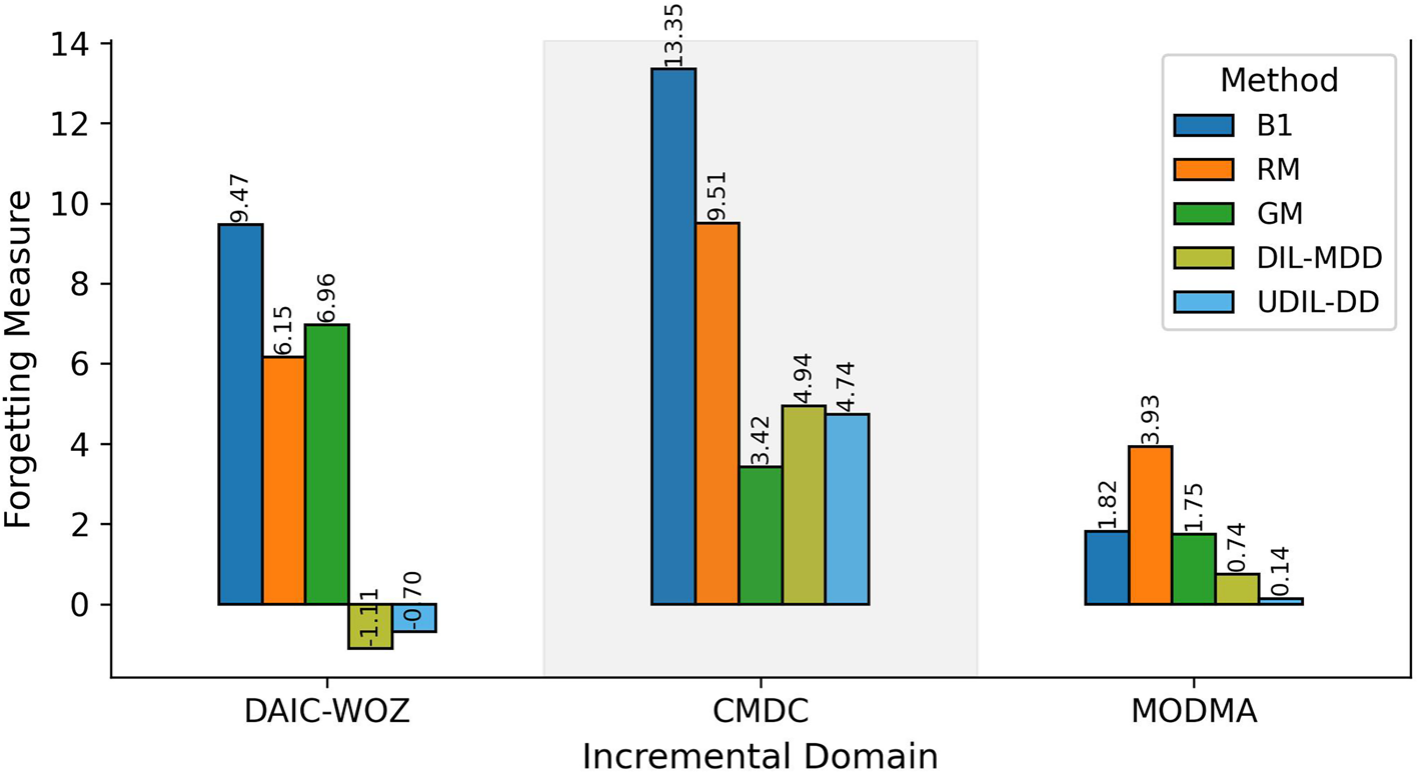
Forgetting analysis across sequential domain-incremental tasks. The score indicates the drop in performance on earlier domains after training on subsequent domains. Lower values indicate better memory retention.

**Fig. 4:**
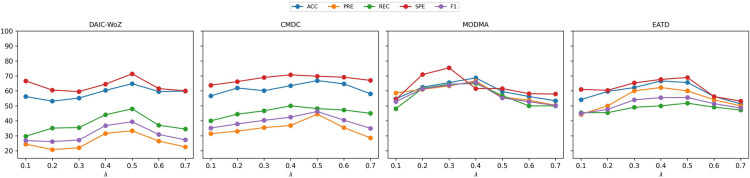
Impact of trade-off hyperparameter $\lambda$ on various metrics across DAIC-WoZ, CMDC, MODMA, and EATD domains.

- *Threshold Setting:* We investigate the effect of different thresholding strategies on classification performance under the continual setup (DAIC-WoZ $\rightarrow$ CMDC $\rightarrow$ MODMA $\rightarrow$ EATD), as reported in Table 2. We compare five fixed thresholds (S1–S5), Adaptive Global Threshold Learning (AGTL, S6), and our proposed Uncertainty-guided Adaptive Class-tailored Threshold Learning (UACTL, S7). Performance shows initial improvement with moderate thresholds; however, it declines at the extremes. For example, S1 ($\tau = 0.30$) and S5 ($\tau = 0.50$) result in increased precision and specificity; however, they experience significant declines in recall and F1 scores (e.g., F1 = 13.75% on DAIC-WoZ in S5), indicating an over-filtering of uncertain samples. S3 ($\tau = 0.40$) demonstrates enhanced balance; however, its improvements are limited across various domains. The adaptive global threshold (AGTL) demonstrates superior performance compared to all fixed settings. AGTL adjusts the threshold across the training distribution, resulting in more consistent outcomes, particularly on MODMA (F1 = 60.93%) and EATD (F1 = 51.87%). Nonetheless, its class-agnostic nature results in imbalanced performance, especially regarding the recall of minority classes. Our proposed UACTL approach outperforms all baseline models by applying uncertainty-aware, class-specific thresholds for enhanced learning outcomes. UACTL attains the highest F1 score across nearly all domains, including DAIC-WoZ (50.49%), CMDC (49.17%), MODMA (63.65%), and EATD (66.35%), demonstrating significant improvements in recall, such as 82.56% on DAIC-WoZ. This demonstrates its capability to address class imbalance and prediction bias by effectively retaining informative, confident samples for each class. To further assess class imbalance, we report Macro-F1 and Balanced Accuracy in Table S2 of the Supplementary Material.
- *Catastrophic Forgetting Analysis:* To investigate the model’s ability to retain knowledge over time, we evaluate the extent of catastrophic forgetting exhibited by all methods across incremental domains DAIC-WoZ $\rightarrow$ CMDC $\rightarrow$ MODMA $\rightarrow$ EATD. Following prior works, we define the forgetting measure as the difference in classification performance on a previously learned domain before and after the model learns a new domain. The larger the drop, the more severe the forgetting. The computed forgetting scores for each method across the DAIC, CMDC, MODMA, and EATD domains are summarized in Figure 3. The results show that baseline methods relying on sequential fine-tuning suffer the most from catastrophic forgetting. Notably, Baseline1 (B1) demonstrates the highest rate of forgetting in the DAIC (9.47%) and CMDC (13.35%) domains. Similarly, RM and GM methods exhibit significant forgetting, particularly in the earlier domains. The observed decline in performance is a result of the model’s inclination to overfit to the most recently trained domain, which leads to the overwriting of previously learned knowledge. In contrast, both DIL-MDD and the proposed UDIL-DD effectively mitigate this issue. In all domains, consistently lower forgetting scores are demonstrated. UDIL-DD exhibits the least amount of forgetting in DAIC ($-0.7$%) and MODMA (0.14%), while maintaining full performance on the final domain (EATD). The results demonstrate UDIL-DD’s capability of retaining prior knowledge even in the absence of access to historical data, attributed to its uncertainty-aware, data-free domain alignment approach. These results emphasise the effectiveness of domain-incremental learning frameworks, particularly in practical applications where data privacy or storage constraints limit access to prior data. UDIL-DD demonstrates strong robustness, showcasing its enhanced capability to generalise across tasks while retaining knowledge, addressing a key challenge in incremental learning for MDD detection.
- *Uncertainty Analysis and Out-of-Distribution (OOD) Detection:* To further evaluate the generalization capability of our method, we analyze the model’s uncertainty behavior and its performance on out-of-distribution (OOD) detection, particularly when encountering an unseen domain. Following standard protocols, we use the trained model (on DAIC-WoZ, CMDC, and MODMA) and measure its predictive uncertainty on both the previously seen domains and the untrained target domain (EATD). As shown in Table 3, the model exhibits a higher uncertainty on EATD (mean = 0.58, std = 0.11) than on previously trained domains (mean = 0.32, std = 0.06). This sharp contrast reflects the model’s capacity to identify unfamiliar data and adjust its confidence accordingly, thus serving as an implicit indicator of newer domains. To evaluate out-of-distribution (OOD) detection capabilities, we consider EATD as the unseen domain and assess the performance of various methods using AUROC and AUPR metrics, as detailed in Table 4. The proposed UDIL-DD attains the highest scores (AUROC = 84.20, AUPR = 81.33), indicating a robust ability to differentiate EATD samples from in-distribution data. Baseline1 demonstrates significantly lower AUROC (74.12) and AUPR (71.08), suggesting a restricted sensitivity to domain shift. Among the competing methods, DIL-MDD performs closest to UDIL-DD but still falls short by approximately 1.75% in AUROC and 1.71% in AUPR. These observations suggest a strong correlation between higher uncertainty on unseen data and improved OOD detection performance. Methods such as UDIL-DD, which incorporate uncertainty modelling into threshold learning and domain alignment, exhibit effective novelty detection and diminished forgetting. This ability highlights the practicality of uncertainty-aware learning for real-world applications, where unforeseen domain shifts are unavoidable, particularly in mental health detection tasks.
- *Hyperparameter analysis:* Further, we conduct a thorough analysis of the influence of the trade-off hyperparameter $\lambda$ on overall model performance, as demonstrated in Figure 4. This hyperparameter regulates the equilibrium between classification loss and domain alignment objectives within the proposed UDIL-DD framework. The experiments are conducted on the incremental learning task DAIC-WoZ $\rightarrow$ CMDC $\rightarrow$ MODMA $\rightarrow$ EATD, with $\lambda$ varying from 0.1 to 0.7. The results indicate a clear pattern: as $\lambda$ increases, performance metrics show an initial improvement followed by a decline across the majority of datasets. This illustrates the common trade-off dynamics: lower values of $\lambda$ minimise the importance of domain alignment, whereas higher values impose excessive restrictions on the model, which could negatively impact classification accuracy. The highest performance is noted at $\lambda = 0.5$, where the model reaches its maximum F1 score on DAIC-WoZ (F1 = 39.34%), CMDC (F1 = 46.23%), and EATD (F1 = 55.61%). This suggests an optimal balance between the contributions of classification and domain alignment losses. The optimal F1 score for MODMA is 65.80%, occurring at $\lambda = 0.4$, indicating that the ideal balance may vary based on the characteristics of the target domain. It is evident that extreme values of $\lambda$, such as 0.1 or 0.7, consistently result in diminished performance. At $\lambda = 0.1$, the DAIC-WoZ F1 score decreases to 26.76%. In contrast, at $\lambda = 0.7$, both CMDC and MODMA exhibit significant reductions, recording scores of 34.99% and 50.18%, respectively. The findings indicate that inadequate balancing of objectives may either hinder domain adaptation or alter class boundaries, resulting in performance instability. Furthermore, these findings emphasise the importance of precisely adjusting $\lambda$ to address different levels of domain complexity. Although $\lambda = 0.5$ provides the strongest generalisation in our context, optimal values can vary between tasks, and choosing this parameter may necessitate further validation efforts. Our results indicate that the UDIL-DD framework demonstrates both sensitivity and stability when subjected to moderately varying $\lambda$ values, which underscores its adaptability in various MDD detection scenarios.

**Table 2:** The ablation experiments of the proposed method conducted under various threshold settings on the DAIC-WoZ $\rightarrow$ CMDC $\rightarrow$ MODMA $\rightarrow$ EATD incremental learning setup.

**Id**	**Threshold**	DAIC-WoZ					CMDC					MODMA					EATD				
		ACC	PRE	REC	SPE	F1	ACC	PRE	REC	SPE	F1	ACC	PRE	REC	SPE	F1	ACC	PRE	REC	SPE	F1
S1	$\tau = 0.30$	**80.58**	65.11	11.11	92.82	18.98	**71.56**	55.64	19.15	**94.45**	28.49	59.24	50.00	33.33	70.54	40.00	64.10	50.00	33.33	71.18	40.00
S2	$\tau = 0.35$	57.73	19.41	27.68	70.15	22.82	61.54	28.57	45.45	68.86	35.09	56.86	56.14	33.33	**78.60**	41.83	56.67	52.14	33.33	73.08	40.67
S3	$\tau = 0.40$	64.88	26.97	48.25	69.17	34.60	64.31	30.15	45.45	72.46	36.25	62.75	60.77	40.27	71.96	48.44	62.59	44.72	40.00	75.21	42.23
S4	$\tau = 0.45$	52.08	10.85	22.22	59.07	14.58	64.31	31.25	28.28	77.24	29.69	62.75	42.07	33.33	72.08	37.19	62.59	33.04	25.48	74.32	28.77
S5	$\tau = 0.50$	80.01	33.33	8.66	92.96	13.75	**71.56**	31.25	6.89	**94.45**	11.29	50.00	45.45	50.00	50.00	47.62	55.90	45.45	50.00	50.00	47.62
S6	AGTL	**80.58**	**67.67**	22.22	**94.15**	33.45	71.06	**55.64**	33.33	85.55	41.69	61.55	60.33	**61.55**	69.23	60.93	**69.55**	55.00	49.07	**76.10**	51.87
S7	UACTL	75.19	36.36	**82.56**	82.05	**50.49**	67.90	43.68	**56.25**	73.01	**49.17**	**66.71**	**61.68**	65.76	66.01	**63.65**	67.13	**66.68**	**66.03**	66.01	**66.35**

**Table 3:** Predictive uncertainty statistics across in-distribution and out-of-distribution (OOD) domains.

Domain	Mean uncertainty	Std uncertainty
Trained (DAIC-WoZ + CMDC + MODMA)	0.32	0.06
EATD (Untrained)	0.58	0.11

The model is trained on DAIC-WoZ, CMDC, and MODMA, and evaluated on EATD as an unseen target.

**Table 4:** OOD detection performance when EATD is considered as the out-of-distribution (OOD) domain.

Method	AUROC	AUPR
Baseline1	74.12	71.08
RM-based	78.30	75.44
GM-based	80.10	77.85
DIL-MDD	82.45	79.62
**UDIL-DD**	**84.20**	**81.33**

Metrics include AUROC and AUPR, evaluated using the uncertainty scores produced by each method.

### Limitations and future work

While the proposed UDIL-DD framework exhibits strong performance in continual cross-domain MDD detection, certain limitations necessitate further exploration. The implementation of uncertainty-guided thresholding and data-free domain alignment effectively addresses issues such as class imbalance and catastrophic forgetting; nevertheless, the existing framework assumes a sequential domain adaptation process. This design, although practical, may struggle to generalise in more dynamic clinical scenarios where domains arrive asynchronously or necessitate reordering. Furthermore, this study focuses primarily on clinical transcripts, which, while useful, may not capture the full spectrum of depressive symptoms. In practice, MDD diagnosis often benefits from multimodal signals such as audio, video, and physiological data (e.g., EEG), which offer richer context and potentially greater predictive power. The integration of these modalities presents several challenges, such as differences in data acquisition protocols and the need for effective cross-modal alignment strategies. Moreover, although evidential uncertainty improves threshold learning, it relies on Dirichlet-based assumptions, which may be limited in modelling more complex uncertainty patterns, particularly in high-dimensional multimodal settings.

In the future work, UDIL-DD can be extented to handle multimodal inputs and improve uncertainty interpretability, thus leveraging the complementary strengths of various clinical signals. Another potential future direction involves the integration of federated learning to facilitate collaborative model training among various health institutions while maintaining data privacy, thereby utilising diverse clinical data resources without the need to share raw data. Additionally, investigating the integration of Internet of Things (IoT) based platforms for remote health monitoring and diagnosis may facilitate real-time mental health interventions via mobile or wearable devices. Furthermore, future studies should explore incorporating temporal progression to analyze the dynamic evolution of feature representations, further enhancing continuous model adaptation in time-varying clinical environments.

## Conclusion

This paper proposes UDIL-DD, a framework designed for uncertainty-aware domain-incremental learning specifically aimed at detecting MDD through text analysis. The proposed method incorporates evidential deep learning to achieve robust uncertainty modelling and utilises data-free domain alignment to maintain historical knowledge while adhering to privacy constraints. The experimental findings from multiple MDD benchmark datasets indicate that UDIL-DD successfully adapts to domain shifts, addresses class imbalance, and sustains strong performance across sequential learning phases. In practice, this framework could allow clinicians and researchers to incrementally update MDD detection models as new data emerges, without compromising patient privacy or prior knowledge. By explicitly quantifying predictive uncertainty, our framework enables more reliable and interpretable decisions, which are essential for early intervention and personalized mental health care. Future research may investigate federated learning and multimodal extensions that incorporate additional features, including visual and physiological signals, to improve detection accuracy. Further, we plan to investigate advanced generative or self-supervised approaches and extend this framework to other psychiatric or psychological conditions.

## Supplementary Information

Supplementary Information.

## Data Availability

The datasets used in this study are available for academic research purposes as follows: Chinese Multi-modal Depression Corpus (CMDC): Accessible upon request through https://ieee-dataport.org/open-access/chinese-multimodal-depression-corpus. DAIC-WOZ Dataset: Accessible upon request through https://dcapswoz.ict.usc.edu/. Emotional Audio-Textual Depression Corpus (EATD)-Corpus: Publicly available via GitHub at https://github.com/speechandlanguageprocessing/ICASSP2022-Depression. Multi-modal Open Dataset for Mental-disorder Analysis (MODMA): Publicly accessible at https://modma.lzu.edu.cn/data/index/ upon signing a standard End User License Agreement (EULA). All datasets were used in accordance with their respective access guidelines and terms.
