# Supplementary material for: Uncertainty aware domain incremental learning for cross domain depression detection
Source: Sci Rep. 2025 Jul 14;15:25344. doi: 10.1038/s41598-025-10917-y (PMC12260041; doi:10.1038/s41598-025-10917-y)
Supplement: Supplementary file 1 — Supplementary Information. [file 41598_2025_10917_MOESM1_ESM.pdf]

Uncertainty Aware Domain Incremental Learning for Cross Domain Depression Detection

Zita Lifelo[1], Jianguo Ding[2,*], Huansheng Ning[1], and Sahraoui Dhelim[3]

[1]University of Science and Technology Beijing, School of Computer and Communications Engineering, Beijing, 100083, China
[2]Blekinge Institute of Technology, Department of Computer Science, 371 79 Karlskrona, Sweden
[3]Dublin City University, School of Computing, Dublin 9, Ireland
[*]jianguo.ding@bth.se

Supplementary Material

Performance Evaluation

To further evaluate the effectiveness of the proposed method under class imbalance, we report Macro-F1 and Balanced Accuracy (Bal-ACC) metrics for each domain across four domain-incremental tasks, as detailed in Table S1. These metrics offer additional insights that extend beyond precision, recall, and specificity, particularly when assessing per-class fairness in imbalanced settings.UDIL-DD consistently demonstrates superior performance across all tasks and domains, demonstrating its robustness to class imbalance. For example, UDIL-DD performs well across majority and minority classes in Task #1, achieving the highest Macro-F1 scores across all domains, including CMDC (67.93%) and DAIC-WoZ (68.29%). Similarly, the balanced accuracy scores show that UDIL-DD outperformed all baselines, reaching 70.46% on CMDC and 70.77% on DAIC-WoZ.

UDIL-DD continues to make steady progress in Task #2, especially on EATD, where it attains a Macro-F1 of 67.18% and Bal-ACC of 66.02%. Similar patterns are shown in Tasks #3 and #4, where UDIL-DD performs better on the majority of domains than DIL-MDD and all baselines. In Task #3, for example, UDIL-DD performs well on EATD (56.23%) and MODMA (67.25%), and it also obtains the highest Macro-F1 on CMDC (63.39%). Notably, certain approaches (such as GM-based on DAIC-WoZ) perform badly in Macro-F1, indicating weak minority class sensitivity, even while they attain good balanced accuracy by favouring the majority class. These results support the claim that UDIL-DD achieves more balanced learning across classes in various incremental domains instead of favouring dominant patterns in the data, which is important for generalisation and fairness in mental health prediction tasks.

Method	CMDC		DAIC-WoZ		EATD		MODMA	
	Macro-F1	Bal-ACC	Macro-F1	Bal-ACC	Macro-F1	Bal-ACC	Macro-F1	Bal-ACC
Baseline1	30.18	45.20	47.36	53.59	34.17	58.51	33.28	55.48
RM-based	46.78	57.46	60.66	64.71	33.33	54.96	25.07	52.27
GM-based	51.09	61.05	53.07	59.37	33.33	54.79	22.64	59.37
DIL-MDD	64.71	66.57	66.75	68.09	46.90	62.80	46.16	60.58
UDIL-DD	**67.93**	**70.46**	**68.29**	**70.77**	**47.99**	**63.96**	**51.76**	**60.59**

(a) Task #1: CMDC → DAIC-WoZ → EATD → MODMA

Method	DAIC-WoZ		CMDC		MODMA		EATD	
	Macro-F1	Bal-ACC	Macro-F1	Bal-ACC	Macro-F1	Bal-ACC	Macro-F1	Bal-ACC
Baseline1	23.94	55.28	22.67	45.00	47.55	54.39	39.33	52.97
RM-based	32.35	55.68	40.08	53.92	51.66	53.70	45.62	53.93
GM-based	47.45	62.75	47.81	59.65	45.58	54.19	45.23	54.52
DIL-MDD	45.45	81.42	49.22	62.36	64.24	64.85	66.96	65.73
UDIL-DD	**56.27**	**82.31**	**51.09**	**64.63**	**64.83**	**65.89**	**67.18**	**66.02**

(b) Task #2: DAIC-WoZ → CMDC → MODMA → EATD

Method	EATD		MODMA		CMDC		DAIC-WoZ	
	Macro-F1	Bal-ACC	Macro-F1	Bal-ACC	Macro-F1	Bal-ACC	Macro-F1	Bal-ACC
Baseline1	37.16	50.56	54.05	58.95	39.98	58.27	25.83	56.57
RM-based	55.88	62.75	**68.19**	**69.30**	37.33	56.09	29.61	57.89
GM-based	48.52	63.08	59.66	61.08	26.51	53.58	28.46	51.74
DIL-MDD	54.60	63.45	67.59	64.49	60.47	61.51	45.57	58.63
UDIL-DD	**56.23**	**64.15**	67.25	65.20	**63.39**	**61.29**	**47.67**	**58.10**

(c) Task #3: EATD → MODMA → CMDC → DAIC-WoZ

Method	MODMA		EATD		DAIC-WoZ		CMDC	
	Macro-F1	Bal-ACC	Macro-F1	Bal-ACC	Macro-F1	Bal-ACC	Macro-F1	Bal-ACC
Baseline1	47.02	53.26	29.85	55.15	27.87	44.87	25.92	51.30
RM-based	56.95	57.38	21.70	51.09	33.36	48.56	37.42	49.24
GM-based	54.63	61.95	39.62	62.99	38.94	**68.44**	44.44	61.43
DIL-MDD	65.04	63.38	50.35	**65.70**	64.46	65.65	57.66	60.13
UDIL-DD	**66.15**	**64.79**	**50.86**	64.94	**64.78**	65.85	**58.49**	**62.06**

(d) Task #4: MODMA → EATD → DAIC-WoZ → CMDC

Table S1. Performance comparison across four domain-incremental tasks using various methods. Metrics reported are Macro-F1 and Balanced Accuracy (Bal-ACC).

Performance Comparison with Domain Adaptation Methods

We conduct additional experiments to evaluate the robustness of our proposed method using MODMA as the source domain. This supports our previous experiments that primarily utilised DAIC-WoZ as the starting point. We assess performance in three domain-adaptation tasks: (i) MODMA → DAIC-WoZ, (ii) MODMA → CMDC, and (iii) MODMA → EATD, and report the results in Figure S1.

In all three tasks, UDIL-DD exhibits consistently competitive and balanced performance. In Task #1, UDIL-DD attains F1 scores of 49.49% on MODMA and 58.69% on DAIC-WoZ, surpassing the performance of baseline methods, including DIL-MDD. In Task #2, UDIL-DD achieves the highest F1 scores of 59.72% on MODMA and 57.76% on CMDC. In Task #3, UDIL-DD exhibits a similar pattern, maintaining performance stability with F1 scores of 45.56% on EATD and 55.16% on

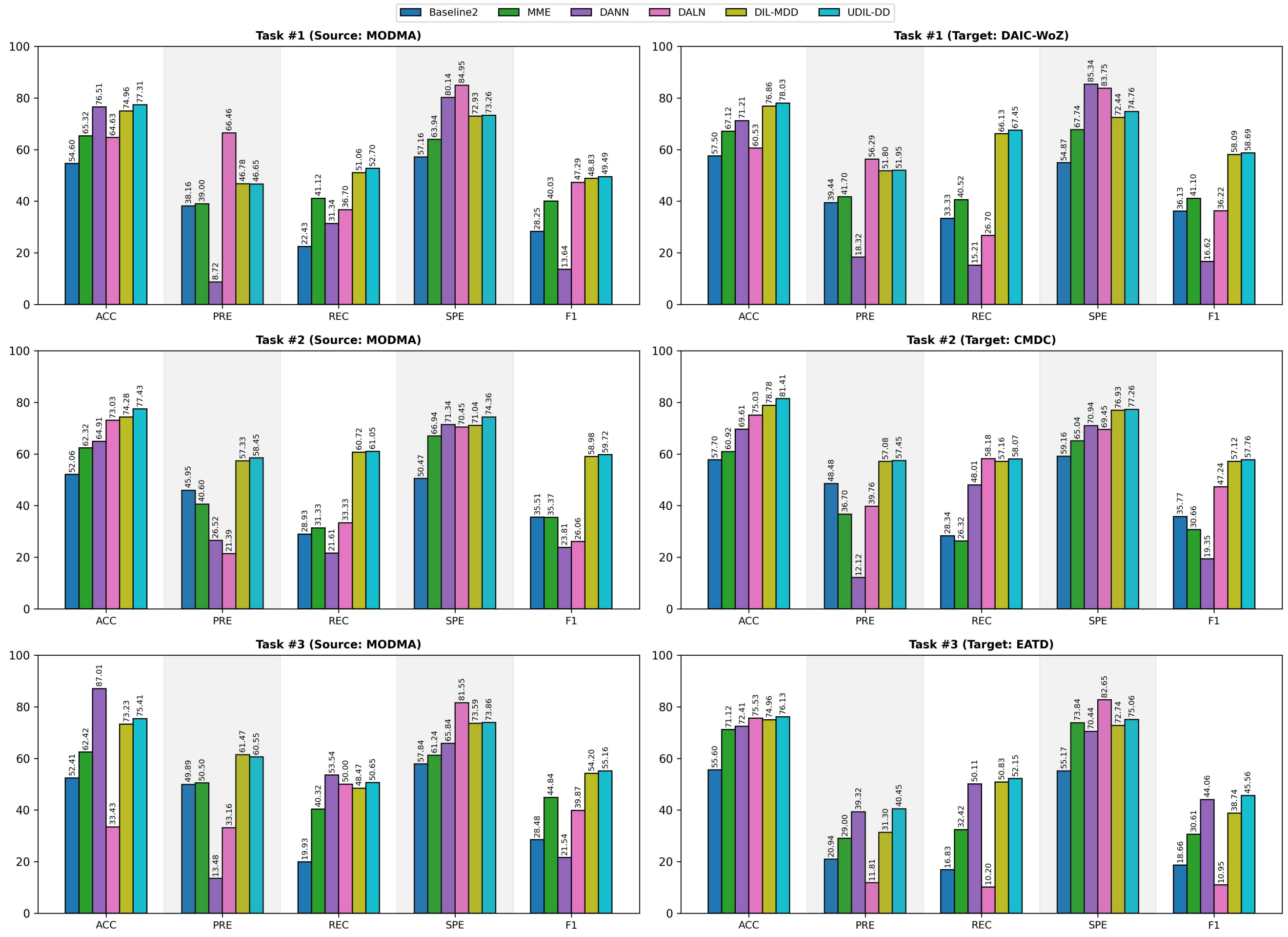

Figure S1. Comparative performance of domain adaptation (DA) and domain-incremental learning (DIL) methods across source and target domains.

MODMA.

Among the DA baselines, DALN demonstrates strong performance in certain metrics, such as precision and specificity; however, it exhibits low recall, resulting in imbalanced outcomes. For instance, in Task #1, DALN achieves a specificity of 84.95% on MODMA, yet its recall is only 36.70%. DANN demonstrates high accuracy in Task #3; however, it shows instability in precision and recall, indicating a tendency for overfitting to the target domain. MME demonstrates greater stability but provides only slight enhancements compared to Baseline2 and does not possess the necessary generalisation for effective source-target transfer. DIL-MDD exhibits the most robust baseline overall, delivering balanced performance across various domains; nonetheless, it is consistently outperformed by UDIL-DD. These results demonstrate the benefits of integrating uncertainty-aware sample filtering and class-specific threshold learning.

Threshold Setting Ablation

We further evaluate the effectiveness of our uncertainty-guided threshold learning (UACTL) strategy by analysing Macro-F1 and Bal-ACC under different threshold settings. As shown in Table S2, fixed threshold configurations (e.g., $\tau = 0.30$ or $\tau = 0.50$) lead to poor Macro-F1 despite sometimes achieving high specificity, indicating over-filtering or under-sensitivity to the minority class.

Id	Threshold	DAIC-WoZ		CMDC		MODMA		EATD	
		Macro-F1	Bal-ACC	Macro-F1	Bal-ACC	Macro-F1	Bal-ACC	Macro-F1	Bal-ACC
S1	$\tau = 0.30$	19.96	51.96	28.47	56.80	40.27	51.94	39.59	52.26
S2	$\tau = 0.35$	26.48	48.92	36.33	57.16	42.21	55.96	40.87	53.21
S3	$\tau = 0.40$	31.88	58.71	36.36	58.96	47.20	56.11	45.72	57.61
S4	$\tau = 0.45$	16.83	40.64	31.47	52.76	44.64	52.71	31.55	49.90
S5	$\tau = 0.50$	14.15	50.81	13.87	50.67	48.81	50.00	50.00	50.00
S6	AGTL	43.08	58.19	43.62	59.44	62.08	65.39	53.98	62.58
S7	UACTL	**66.27**	**82.31**	**49.09**	**64.63**	**64.67**	**65.89**	**66.18**	**66.02**

Table S2. Ablation experiments of the proposed method under various threshold settings on the DAIC-WoZ $\rightarrow$ CMDC $\rightarrow$ MODMA $\rightarrow$ EATD setup. Metrics reported are Macro-F1 and Balanced Accuracy (Bal-ACC).

AGTL improves overall performance but remains class-agnostic, which results in uneven performance across domains (e.g., EATD Macro-F1 = 53.98%). In contrast, UACTL achieves the highest Macro-F1 and Bal-ACC on all domains. These results confirm that our uncertainty-aware, class-specific thresholding mechanism is highly effective in alleviating class imbalance. By incorporating both divergence and predictive uncertainty, UACTL ensures that only confident and class-representative samples guide learning, leading to better generalisation and fairer performance.
